# Etymologia: Cholera

**DOI:** 10.3201/eid1711.ET1711

**Published:** 2011-11

**Authors:** Nancy Männikkö

**Keywords:** cholera, etymologia, bile, Galen, four humors, Regimen Sanitatis Salernitanum, history

## [käl′ər-ə]

From the Greek *cholē* for bile. Although the term cholera is now used only to refer to disease caused by the bacterium *Vibrio cholerae*, until the late 19th century any diarrheal illness might be referred to as cholera. For many centuries, medicine in Europe was based on Galen’s theory of the 4 humors in the body: blood, bile, black bile, and phlegm. Diarrhea and vomiting were interpreted as the body’s attempt to restore balance and good health by expelling excess choler, hence, many gastroenterological illnesses were referred to as cholera. A 12th century treatise, the Regimen Sanitatis Salernitanum, described the effects of excess choler thusly, “Your tongue will seem all rough, and oftentimes cause vomits, unaccustomed and hateful, great thirst, your excrements are full of slime, the stomach squeamish, sustenance ungrateful, your appetite will seem in nought delighting.”Sources: Dorland’s illustrated medical dictionary. 31st ed. Philadelphia: Saunders; 2007; The Four Temperaments [cited 2011 Aug 19]. http://www.fisheaters.com/fourtemperaments.html; Howard-Jones N. Choleranomalies: the unhistory of medicine as exemplified by cholera. Perspect Biol Med. 1972;15:422–33. PubMed; Oxford dictionaries online. 2011 [cited 2011 Aug 19]. http://oxforddictionaries.com/definition/cholera?region=us; Tauxe RV. Cholera. In: Hussler WJ, Sussman M, editors. Topley & Wilson’s microbiology and microbial infections, 9th ed. Vol. 3: Bacterial infections. New York: Oxford University Press; 1998. p. 495–512.

